# Oral Supplementation with Baker's Yeast Beta Glucan Is Associated with Altered Monocytes, T Cells and Cytokines following a Bout of Strenuous Exercise

**DOI:** 10.3389/fphys.2017.00786

**Published:** 2017-10-20

**Authors:** Brian K. McFarlin, Adam S. Venable, Katie C. Carpenter, Andrea L. Henning, Stephan Ogenstad

**Affiliations:** ^1^Applied Physiology Laboratory, Department of KHPR, University of North Texas, Denton, TX, United States; ^2^Department of Biological Sciences, University of North Texas, Denton, TX, United States; ^3^Science Division, Isagenix, Gilbert, AZ, United States; ^4^Statogen Consulting, LLC, Zebulon, NC, United States

**Keywords:** baker's yeast beta glucan, cytokine response, monocytes, T cells, Th1/Th2, exercise response

## Abstract

Exercise and physical labor in extreme environmental conditions causes transient decreases in immune cell and cytokine concentrations, likely increasing the susceptibility to opportunistic infection. Baker's yeast beta glucan (BYBG) has been previously demonstrated to be an effective countermeasure in athletes, but its effectiveness in individuals of average fitness under similar physical stress is unknown. The purpose of this study was to determine if 10 days of oral supplementation with BYBG could modify previously observed suppression of monocytes, T cells, circulating and whole blood LPS-stimulated cytokines due to strenuous exercise. Venous blood samples were collected from 109 healthy volunteers prior to, immediately after, 2 and 4 h post-exercise. Monocyte and T cell concentration, cell-surface receptor expression and serum and LPS-stimulated cytokines were assessed. BYBG significantly (*P* < 0.05) altered total and classic monocyte concentration and expression of CD38, CD80, CD86, TLR2, and TLR4 on monocyte subsets. BYBG also significantly increased CD4+ and CD8+ T cell concentration and the exercise response of CCR7+/CD45RA- central memory (T_CM_) cells. Likewise, BYBG significantly (*P* < 0.05) altered serum IFN-γ and IL-2, and LPS-stimulated IFN-γ, IL-2, IL-4, and IL-7. Taken together these data support the hypothesis that oral BYBG supplementation modulates the expected exercise response for individuals of average fitness. This may result in a decrease in susceptibility to opportunistic infections after strenuous exercise.

## Introduction

Our laboratory and others have demonstrated that moderate exercise is beneficial to the immune system. However, strenuous exercise can cause immune suppression for up to 24 h after exercise (McFarlin et al., 2003, 2007a, 2013; Strohacker et al., 2012; Carpenter et al., 2013). This period of transient immunosuppression, often termed the “open window,” represents a time when an individual may be more susceptible to infection (Pedersen and Bruunsgaard, 1995). Our laboratory has sought to use the “open window” paradigm to evaluate the potential of naturally occurring substances to reduce the duration or magnitude of immunosuppression following exercise. One such substance we have studied previously is Baker's yeast beta-glucan (BYBG).

Baker's yeast beta glucan (BYBG) is a known immunomodulator (Pharmacopiea, 2011) that has been studied for over 50 years. However, despite the large body of evidence for this activity, the mechanism of action is not fully characterized. BYBG has been reported to have the capacity to alter a variety of cellular functions, including but not limited to: priming granulocytes for quicker activation, increasing salivary immunoglobulin (Ig)A, improving circulating monocyte count and altering the balance of T helper (Th1/Th2) cytokines (Goodridge et al., 2011; Pharmacopiea, 2011; Qi et al., 2011; Williams et al., 2016; Zheng et al., 2016). Previous research from our laboratory and others into the effects of BYBG on the human immune system following exercise has focused on changes to the cellular immune response and symptoms of cold/flu illness in physically fit individuals under physical stress (such as exercise) (Harger-Domitrovich et al., 2008; Talbott and Talbott, 2009; Carpenter et al., 2013; McFarlin et al., 2013). Indeed, a majority of the published literature has focused on this population. Therefore, it is unknown how these previously reported effects translate to a general population with a much lower fitness level.

In the present study, we have employed a well-characterized model of exercise-induced stress with a study population composed of individuals of average fitness in order to determine if BYBG supplementation mediates a consistent effect on exercise-induced immune system changes regardless of fitness level. In this exercise model, heat exposure is used to increase the physiological stress of a typical exercise session. Thus, combining exercise and heat exposure increases the likelihood of reduced immune response. Specifically, the purpose of the present study was to determine how 10-days of oral supplementation with BYBG alters monocyte concentration, monocyte cell-surface receptor expression, circulating and whole blood LPS-stimulated cytokines and T cell concentration after strenuous exercise. We hypothesized that oral supplementation with BYBG would be associated with improved immune system function in individuals of average fitness both at rest and following a bout of strenuous exercise. The long-term goal of this research is to provide insight concerning the use of functional nutrition to broadly improve human health outcomes.

## Methods

### Experimental design

**Figure 1:**
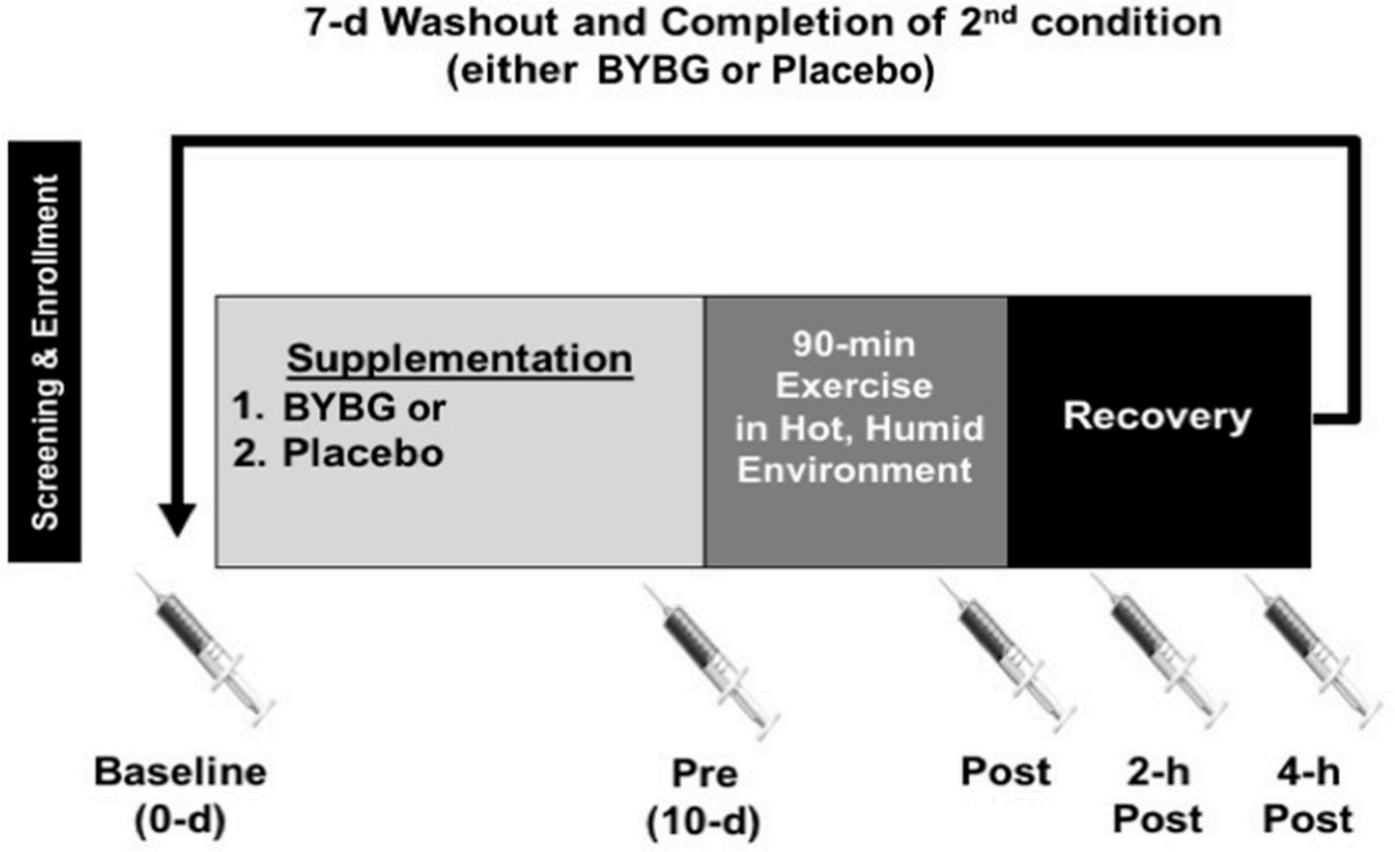
Experimental timeline. After screening, subjects were randomized to an order to complete either a 10-days supplementation with BYBG (250 mg/d) or placebo (rice flour 250 mg/d). After 10-days of supplementation, subjects reported to the laboratory to complete an experimental exercise session (90-min) in a hot, humid environmental chamber. Venous blood samples were collected prior to supplementation (baseline), prior to exercise (Pre), within 5-min of the end of exercise (Post), 2- and 4-h post-exercise. The later three samples were used to assess the effect of exercise on early phase immunity. After collection of the 4-h sample, subjects completed a 7-days washout and repeated the entire protocol again.

All methods described in this study were completed in accordance with the latest *Declaration of Helsinki*. The study was conducted at two locations where Dr. McFarlin worked and thus was approved by both the University of Houston and the University of North Texas Institutional Review Boards (IRB). All subjects gave written and verbal consent to participate. This study was completed in collaboration with an industrial partner and conducted according to previously published guidelines designed to eliminate bias in industry-funded research (Rowe et al., 2009). The study consisted of two, 10-days supplementation periods that were separated by a 7-days washout period (Figure 1). The suitability of the washout period length was based on previously published studies from our laboratory (Carpenter et al., 2013; McFarlin et al., 2013). Blood samples were collected prior to supplementation (baseline), after 10-days of supplementation (pre), within 5-min (min) of completing exercise (post), 2-h after exercise (2-h), and 4-h after exercise (4-h). Subjects reported for all sample collection appointments between 0,400 and 1,000 following an overnight fast (>8 h) and abstention from exercise (>24 h). On the day of the experimental exercise session, subjects maintained a fasting state until collection of the 4-h sample.

### Sample size determination

**Figure 2:**
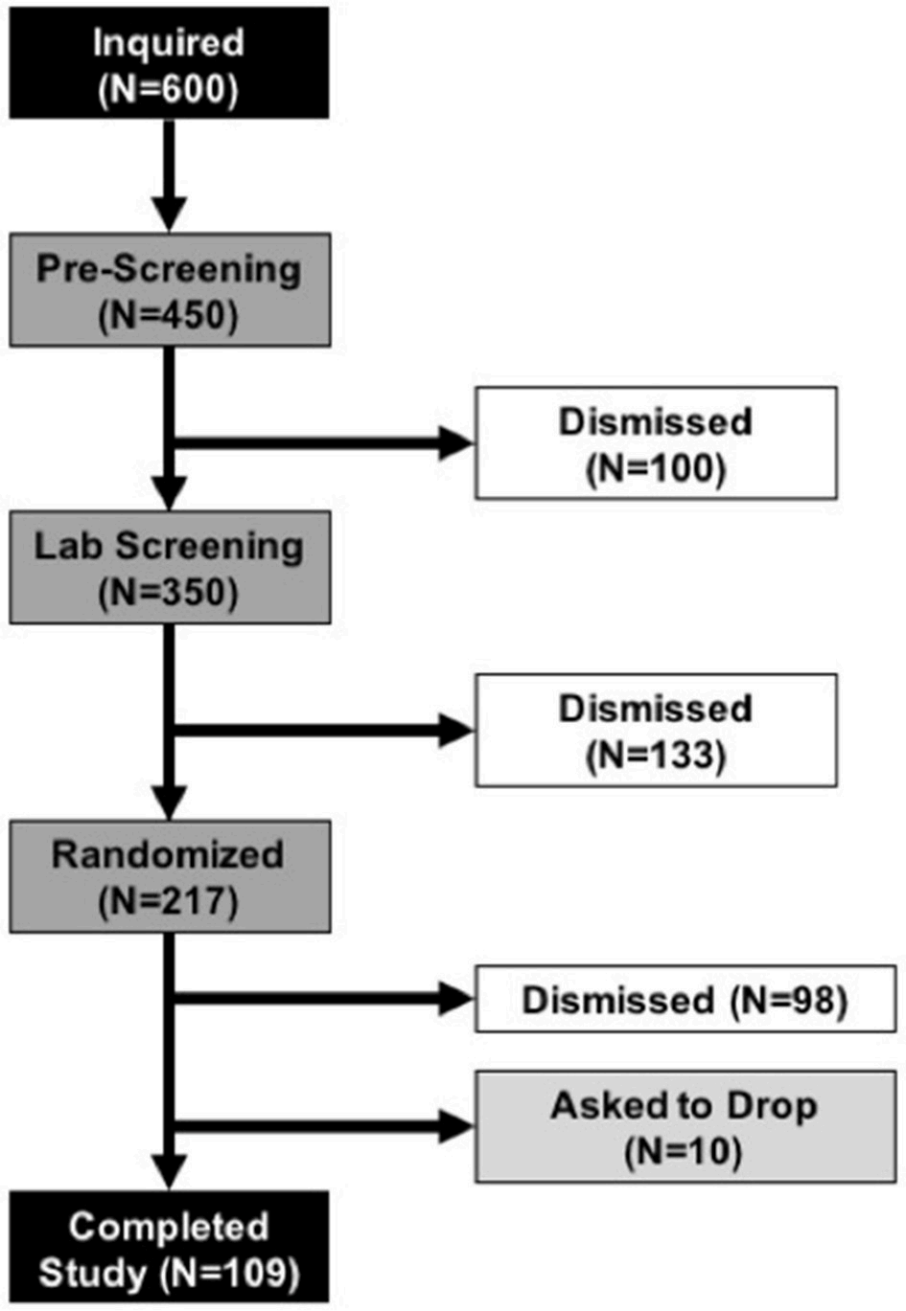
Consort Diagram. Demonstrates subject progression through the various phases of the study from initial inquiry (top black box) to completion of all study tasks (bottom black bar). During the Pre-Screening and Lab Screening phases subjects who did not meet the study requirements were dismissed from further participation. Once subjects were randomized, the most common reasons that subjects were dismissed was for failure to follow the study protocol (i.e., irregular consumption of supplement) or missed study appointments. No subjects dropped or were dismissed due to adverse reactions to the supplement or study protocol.

We previously determined effect sizes associated with BYBG treatment in very active individuals (Carpenter et al., 2013; McFarlin et al., 2013). In general, most effect sizes for monocyte and cytokine changes were of moderate size and reached statistical significance with *n* = 60 subjects. Therefore, in order to confirm effect size calculations in a general population we conducted a pilot study, identical to the study design described below, with 40 enrolled subjects. We observed an unexpectedly high attrition rate (55%) in this study. The most common reason the subjects discontinued participation was due to missed study appointments. No subjects were dismissed due to complications associated with either BYBG or the exercise protocol. Twenty-two subjects completed the pilot and analysis of the data revealed that the smallest effect size (0.19) was associated with monocyte concentration. We decided to estimate sample size for the larger study using 0.15 to take a conservative approach and ensure a sufficient number of subjects. Sample size was estimated using an *a priori* within factor ANOVA test and an effect size of 0.15 (G-Power v.3.1; Dusseldorf, Germany). We determined that a properly powered study required a minimum of 90 individuals to complete the protocol. To account for the anticipated attrition we randomized 217 subjects, 109 of whom completed all the study requirements. The most common reason that subjects were dismissed from the study after randomization was for either failure to follow study protocol (i.e., not taking their supplement) or missed study appointments. A full consort diagram has been provided to detail subject recruitment, enrollment, randomization, and progression in the study (Figure 2).

### Subjects

As described above, determination of the number of subjects to be enrolled in the present study was based on a combination of data from other published studies in our laboratory and a pilot study specific to the subject population and exercise protocol (McFarlin et al., 2006, 2013; Strohacker et al., 2012; Carpenter et al., 2013). Over a period of 2 years, we enrolled subjects until we reached our target of over 100 total subjects completing the entire study protocol. College-aged men and women who were of average physical activity status and who were not actively exercising were recruited. Subjects were screened for inclusion/exclusion criteria using a written medical history form, a whole body DXA scan for body composition (GE Lunar Prodigy; USA), and a graded exercise test on a treadmill. Detailed subject characteristics are presented in Table 1.

**Table 1:** Subject characteristics.

**Characteristic**	**Men (*N* = 50)**	**Women (*N* = 59)**
Age (y)	22 ± 3	21 ± 3
Height (m)	1.74 ± 0.04	1.63 ± 0.07
Weight (kg)	76.98 ± 2.04	62.90 ± 1.74
BMI (kg/m2)	24.74 ± 0.40	23.29 ± 0.53
VO2max (ml/kg/min)	38.3 ± 0.7	36.5 ± 0.7
% Body Fat	17.6 ± 0.9	30.8 ± 1.0

*Values represent the mean ± SEM. No significant gender differences were noted*.

### Dietary supplement conditions

All subjects completed two conditions (placebo and BYBG) using a double-blind, crossover approach with a 7-days washout between conditions. The laboratory team was blinded until all raw data had been analyzed. Placebo consisted of veggie capsules filled with rice flour. BYBG (250 mg/days) was supplied by Kerry Inc. (Beloit, WI, USA). To our knowledge the blinding procedures were sufficient because neither subjects nor investigators were aware of the conditions. Subjects were asked to consume their supplements with food and immediately report any adverse reactions. Subjects did not report any adverse reactions with either placebo or BYBG supplementation in this study, consistent with previous reports from our lab and others (Harger-Domitrovich et al., 2008; Talbott and Talbott, 2009; Auinger et al., 2013; Carpenter et al., 2013; McFarlin et al., 2013).

### Experimental exercise challenge

Subjects reported to the laboratory between 0,400 and 1,000 following an overnight fast (>8-h) and abstention from formal exercise (>24-h). Following collection of the pre-exercise blood sample, subjects were fitted with a heart rate monitor and moved to an environmental chamber set to a heat index of 51 ± 2°C. Subjects completed 6 slow speed (8-min; men = 3 miles per hour (MPH), 38% of max; women = 2.5 MPH, 36% of max) and fast speed (7-min; men = 4.5 MPH, 56% of max; women = 4.0 MPH, 57% of max) treadmill intervals for a total of 90-min of exercise. Subjects were allowed water *ad libitum* and were encouraged to drink fluid regularly by a member of the study staff. Subjects were also fitted with a forehead temperature monitor to ensure that they did not overheat during the exercise session. In any instance where subjects reported signs or symptoms consistent with heat-related illness the experimental exercise challenge was immediately stopped. Subjects who were unable to complete the 90-min protocol were excluded from further participation in the study.

### Venous blood sample collection and processing

Venous blood was collected and treated with either ethylenediaminetetraacetic acid (EDTA), lithium heparin or a clotting agent in Vacuette blood collection tubes (Griener Bio One; Monroe, NC). EDTA blood was used immediately (within 2-h of collection) to isolate peripheral blood mononuclear cells (PBMC) using Histopaque 1,077 (MilliporeSigma; St. Louis, MO) and Leukosep isolation tubes (Griener Bio One). Lithium heparin blood was used immediately (within 2-h of collection) for *in vitro* LPS-stimulation assays. Blood exposed to the clotting agent was allowed to clot at room temperature for 30-min and then centrifuged to yield serum. Isolated serum was frozen at –80°C until analysis for serum cytokines.

### Flow cytometry staining and analysis

All PBMC isolation and staining procedures were completed in a manner consistent with previously established best practice methods in our laboratory (McFarlin et al., 2004, 2006, 2015; Breslin et al., 2012; Strohacker et al., 2012; Carpenter et al., 2013). After isolation of PBMC, cells were checked for viability and concentration using the ViaCount assay per manufacturer instructions (MilliporeSigma, Heyward, CA). All PBMC isolations were >98% viable. All cell fractions were adjusted to 5 × 10^6^/mL viable PBMCs using staining buffer (Affymetrix/eBioscience; San Diego, CA). An aliquot of the PBMC fraction (75 μL) was dispensed into each of 8 library tubes using a calibrated, electronic pipet (Integra Biosciences; Hudson, NH). The first two tubes were used to stain for positive events and the remaining six tubes were used as either fluorescent minus one (FMO) or cells only (negative or auto-fluorescence) controls. A fixable viability dye was used to confirm that all cells were >99% live at the time of staining. Table 2 identifies the relevant product information for each antibody used in the present study. A dump channel consisting of CD19, CD56, viability dye and CD3 (for monocyte tubes) or CD14 (for T-cell tubes) depending on the cell type under investigation was utilized to ensure identification of live cells only of the intended lineage. CD14 and CD16 were used to establish primary and secondary gates for total and monocyte subsets. Primary and secondary gates for T-cells were based on co-expression of CD3 and CD4 (helper T-cell) or CD8 (cytotoxic T-cell). Lineage of T-cells was separately subdivided using CCR7 and CD45RA expression. Once the desired lineage subset was identified, populations were gated to determine CD38, CD80, CD86, TLR2, TLR4, CX3CR1, CD54, and CD195 expression (Table 2). Samples were acquired uncompensated using an EasyCyte 8HT flow cytometer (MilliporeSigma) equipped with 488 and 642 nm lasers. An autosampler was used to automate acquisition and throughput. The staining conditions were optimized to ensure that a minimum of 40,000 PBMC events were collected on each sample.

**Table 2:** Antibodies for flow cytometry staining.

**Target**	**Clone/Catalog #**	**Color**	**Target**	**Clone/Catalog #**	**Color**
CD14	61D3 47-0149-42	APC-eFluor780	CD3	OKT3 17-0037-42	APC
CD16	eBioCB16 25-0168-42	PE-Cy7	CD4	RPA-T4 47-0049-42	APC-eFluor780
CD38	HIT2 17-0389-42	APC	CD8	RPA-T8 45-0088-42	PerCP-Cy5.5
CD80	2D10.4 11-0809-42	FITC	CCR7	3CD12 12-1979-42	PE
CD86	IT2.2 15-0869-42	PE-Cy5	CD45RA	HI100 25-0458-42	PE-Cy7
TLR2	TL2.2 17-9922-42	APC	CD56	CMSSB 47-0567-42	APC-eFluor780
TLR4	HTA125 12-9917-42	PE	CX3CR1	2A9-1 12-6099-42	PE
Fixable viability dye	N/A 65-0865-18	eFluor780	CD54	RR1/1 BMS108FI	FITC
CD19	HIB19 47-0199-42	APC-eFluor780	CD195	313715 HEK/1/85a	PerCP-Cy5.5

After collection, a compensation matrix was created using the various controls and applied to each positively stained sample using commercially available software (FCS Express v.5; De Novo Software; Glendale, CA). For monocyte analysis, primary gates were established to identify total (CD14+), classic (CD14+/CD16−), and non-classic (CD14+/16+) monocytes in a manner consistent with previously published studies from our laboratory (McFarlin et al., 2004, 2006; Strohacker et al., 2012; Carpenter et al., 2013). FMO controls were used to define secondary gates and histograms for the expression of CD38, CD80, CD86 TLR2, TLR4, CX3CR1, CD54, and CD195 on total, classic and non-classic monocytes. For T-cell analysis, primary gates were established to identify total CD3+/CD4+ and CD3+/CD8+ cells and further subdivided based on CCR7 and CD45RA expression. All data files were analyzed in a blinded, coded manner so that investigator bias was minimized. Data were reported as cell concentration, percent positive, or geometric mean fluorescence intensity (gMFI) depending on the outcome measure.

### *In Vitro* cytokine production assay

In order to assess the capacity of blood leukocytes to produce cytokines, we used a method previously validated in our laboratory (McFarlin et al., 2004, 2006; Carpenter et al., 2013). Briefly, lithium heparin-treated whole blood was diluted 1:10 in sterile PBS (pH = 7.4). Aliquots (1 mL) of the diluted blood were transferred to separate wells of a sterile, 24-well tissue culture plate using aseptic technique in a laminar flow tissue culture hood. An aliquot of ultrapure lipopolysaccharide from *Salmonella minnesota* R595 (LPS; final concentration 10 μg/mL; InvivoGen; San Diego, CA) was added to each diluted blood sample (mixing with an electronic pipet set to “pipet and mix” function). This dose of LPS was optimized prior to the study (data not shown). Blood/LPS suspensions were incubated (37°C, 5% CO_2_) for 24-h to elicit cytokine production. After incubation, cell-free supernatants were carefully removed and frozen at −80°C until analysis for cytokine production using a Luminex-based multiplex cytokine assay (MilliporeSigma Milliplex Cat# HSTCMAG28SPMX13; Luminex MagPix; Austin, Texas). Multiplex cytokine assays were completed at the end of the study on all samples at the same time to minimize intra-assay variability. Within this model, we calculated the inter-assay coefficient of variation (CV) to be 8%. Prior to assay, the MagPix was calibrated and verified according to standard Luminex procedures. Each stimulated supernatant was analyzed in duplicate to simultaneously determine the concentration of IFN-γ, IL-1β, IL-2, IL-4, IL-7, TNF-α, IL-10, MCP-3, MIP-1α, and MIP-1β.

### Serum cytokine analysis

At the conclusion of the study all serum samples were thawed and analyzed using a high-sensitivity cytokine assay specifically designed for human serum samples (McFarlin and Venable, 2014). Using commercially-available multiplex technology (MilliporeSigma Milliplex Catalog # HSTCMAG28SPMX13) common to our laboratory (McFarlin and Venable, 2014; McFarlin et al., 2016), we measured each serum sample in duplicate to determine the concentration of IFN-γ, IL-1β, IL-2, IL-4, IL-7, and TNF-α. The same MagPix setup procedures and variability were observed as described above in the measurement of stimulated cytokines.

### Statistical analysis

A mixed linear effects model was used for the analysis, which is a generalization of the standard linear model. The generalization being that the data are permitted to exhibit correlation and nonconstant variability. Both a full and a reduced mixed effects model for crossover design were applied using proc mixed in SAS version 9.3. The full model was used to test for suitability of the wash-out period between BYBG and placebo conditions. With the exception of serum IL-2, no “carry-over” effects were observed from the first to the second period for any other variables and thus statistical analysis is reported as the reduced mixed model analysis. In the case of serum IL-2, additional *post-hoc* testing did not reveal any “carry-over” effect, but the full model provided a better fit of the data than the reduced model. Thus, for serum IL-2, we reported statistical results associated with the full mixed linear model. In these models the class variables were treatment, sequence, the repeated measure (for Baseline, Post, 2- and 4-h), and subject. The denominator degrees of freedom were set to the Kenward and Roger option. The repeated statement had all interactions in the full model and treatment by repeated measures in the reduced model. The significance levels were set to 0.050.

## Results

### Monocyte variables

**Figure 3:**
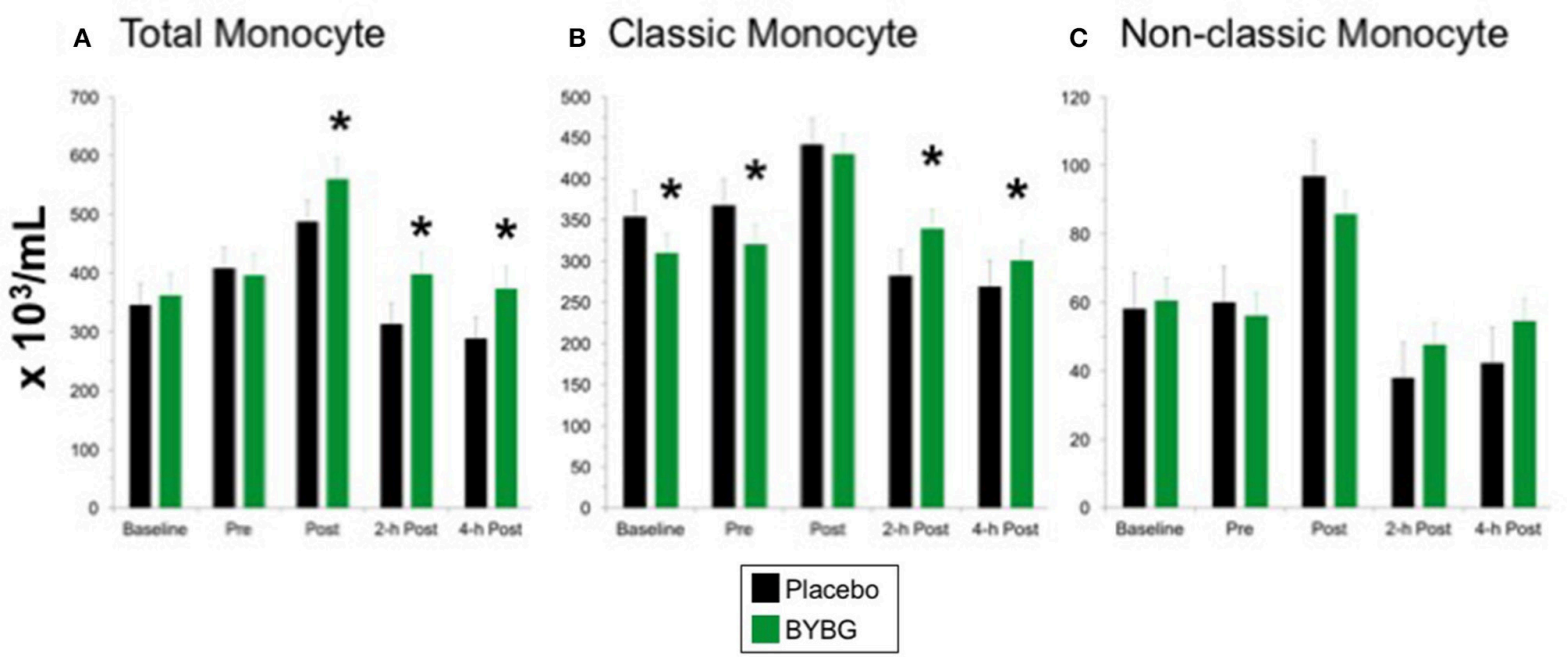
Effect of BYBG on CD14+ cells in peripheral blood. Monocyte concentrations were determined using peripheral blood mononuclear cells (PBMC) isolated from human whole blood samples. PBMCs were analyzed using flow cytometry to determine total (**A**, CD14+), classic (**B**, CD14+/CD16-), and non-classic (**C**, CD14+/CD16+) monocyte concentrations. Placebo (black bars) and BYBG (green bars) are represented separately and were statistically compared. Values represent the mean ± SEM. ^*^indicates BYBG significantly different than placebo (*p* < 0.05).

Strenuous exercise is known to transiently increase the concentration of circulating monocytes during and immediately after exercise (Strohacker et al., 2012; Carpenter et al., 2013). In a typical exercise response monocyte numbers decline in the first 12 h of recovery from exercise and return to baseline levels by 24-h (Walsh et al., 2011). BYBG supplementation increased the concentration of total monocytes found in the peripheral blood after exercise (Post, 2-h, 4-h) compared to placebo but had no effect prior to exercise stress (Figure 3A). The increase in monocyte concentration was due predominantly to an increase in classic monocytes (Figure 3B; CD14+/CD16−) with no change in non-classic monocytes (Figure 3C; CD14+/CD16+) after exercise compared to placebo.

**Figure 4:**
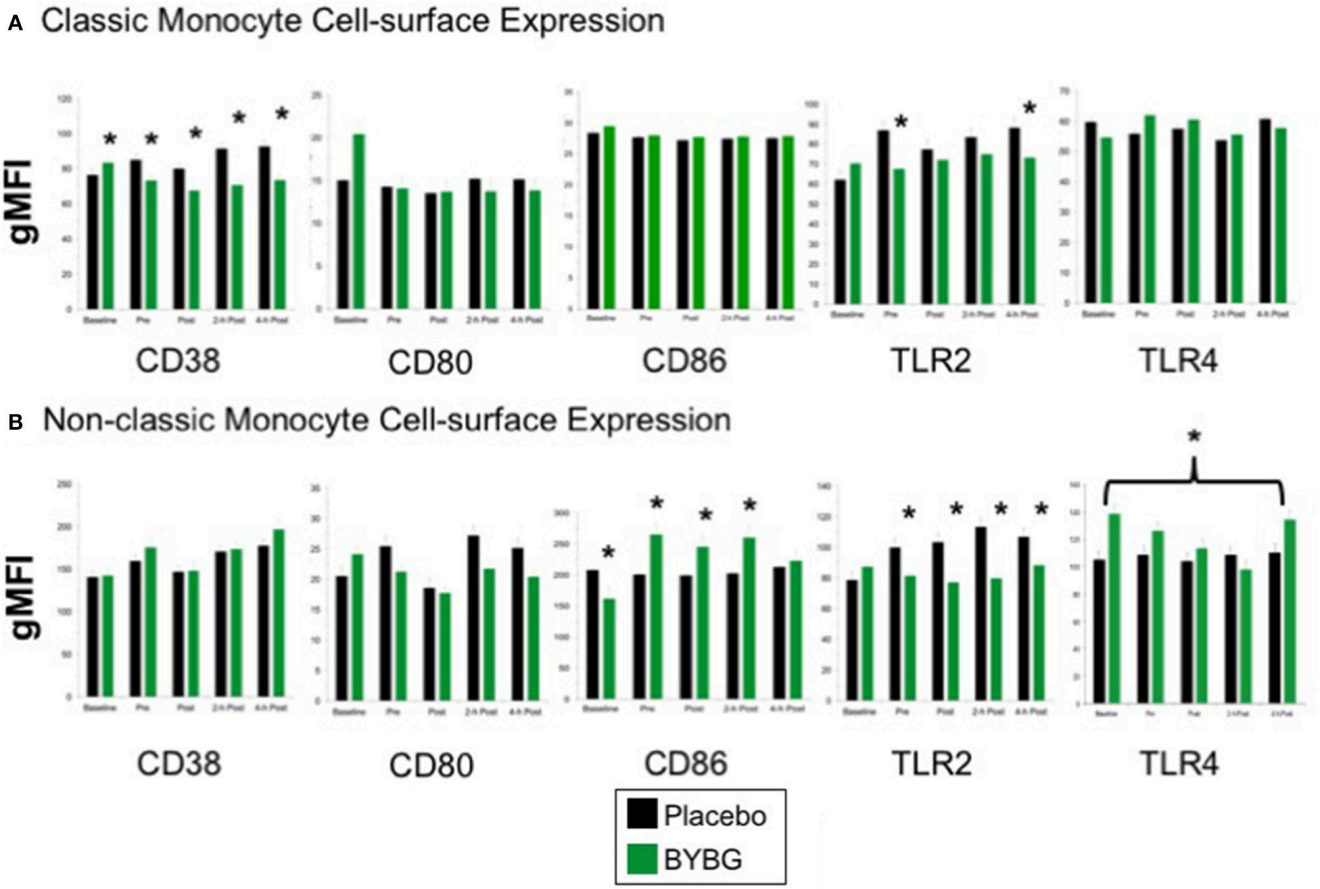
Effect of BYBG on peripheral blood monocyte phenotype. Monocyte cell-surface receptor expression on monocyte subsets was determined using peripheral blood mononuclear cells (PBMC) isolated from human whole blood samples. The expression of CD38, CD80, CD86, TLR2, TLR4 was analyzed by gMFI for each receptor on the whole population of classic CD14+/CD16− **(A)** and non-classic CD14+/CD16+ **(B)**. Values represent the mean ± SEM. ^*^indicates BYBG significantly different than placebo (*p* < 0.05).

Next we investigated whether monocyte phenotype was affected by BYBG supplementation. Surface expression of several proteins known to have roles in canonical monocyte functions were evaluated. CD38 expression was lower on CD14+ cells in the BYBG arm (Figure 4; *p* < 0.0001). All of the modulation of CD38 expression was due to changes in expression on classic monocytes (Figure 4A), no change to CD38 expression was observed on non-classic monocytes (Figure 4B).

When we evaluated expression of the T cell activation co-stimulatory receptors CD80 and CD86, we observed no change in CD80 on any monocyte subset but did observe a significant increase in CD86 expression on non-classic monocytes (Figure 4). TLR2 expression was reduced on both classic and non-classic monocytes with BYBG supplementation. TLR2 expression was down-modulated at all time points after supplementation (pre- through 4-h) on non-classic monocytes while on classic monocytes TLR2 expression was reduced compared to control only at pre-exercise and 4-h post-exercise. In contrast, TLR4 expression was increased on non-classic monocytes. No other monocyte cell surface molecules demonstrated either a significant treatment or interaction effect; however, the traditional exercise response was observed in a manner consistent with previous findings from our laboratory and others (data not shown) (Pedersen and Bruunsgaard, 1995; Mitchell et al., 2002; McFarlin and Mitchell, 2003; McFarlin et al., 2004; Simpson et al., 2009; Carpenter et al., 2013).

### T cells

The response of T-cells to a bout of strenuous exercise is similar to that observed for monocytes and is characterized by a transient increase in concentration immediately after exercise followed by a period of decline below pre-exercise levels during the recovery period. Peripheral blood T cell counts return to baseline within 12- to 24-h after exercise (Gleeson and Bishop, 2005). CD4+ and CD8+ T cells have both been reported to follow this pattern (Nieman et al., 1994; Turner et al., 2010). Since BYBG supplementation prior to exercise improves the exercise-induced transient decrease in peripheral blood monocyte concentration, we investigated whether BYBG supplementation has similar effects on the reported circulatory pattern of T cells after exercise.

### CD4+ T cells

**Figure 5:**
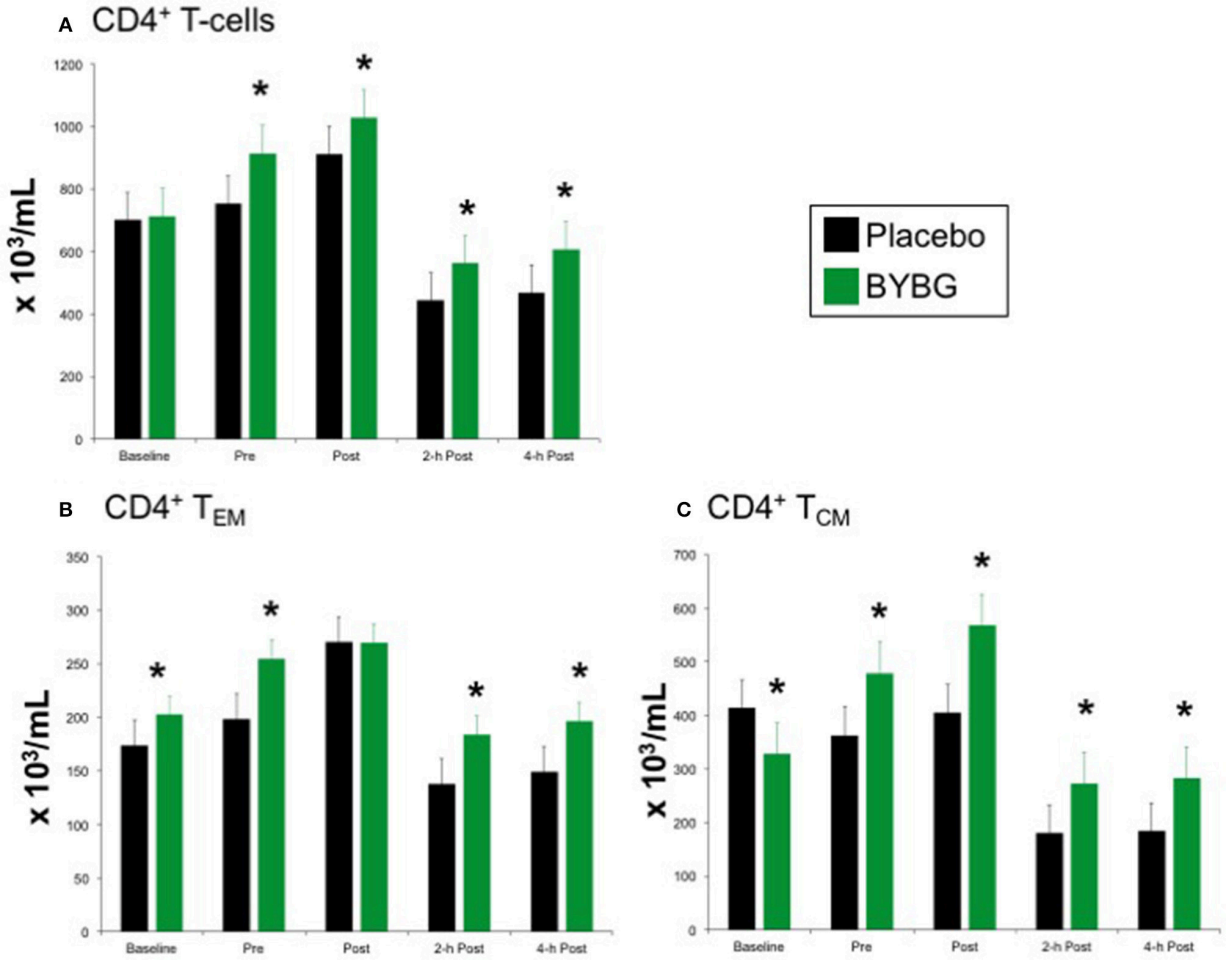
Effect of BYBG on CD4+ T cells. CD4+ T cell concentrations were determined using peripheral blood mononuclear cells (PBMC) isolated from human whole blood samples. PBMCs were analyzed using flow cytometry to determine total (**A**, CD3+/CD4+), T_EM_ (**B**, CCR7-/CD45RA-), and T_CM_ (**C**, CCR7+/CD45RA-) concentrations. Placebo (black bars) and BYBG (green bars) are represented separately and were statistically compared. Values represent the mean ± SEM. ^*^indicates BYBG significantly different than placebo (*p* < 0.05).

The protocol used in this study elicited the expected response of CD4+ T cells to exercise: increased CD4+ cells immediately post-exercise followed by a decrease to below pre-exercise levels that was sustained for at least 4-h (black bars, Figure 5A). Shown in the green bars of Figure 5A, BYBG supplementation had a modest effect on the concentration of total peripheral blood (PB) CD4+ cells. The number of circulating CD4+ cells was significantly increased before exercise (pre) and maintained at a consistent percent greater than placebo through the exercise response (post, 2-h, 4-h). CD4+ is present on multiple distinct populations of T cells present in peripheral blood. Therefore, we used the established convention of differential expression of CCR7 and CD45RA expression to further explore the effects of BYBG supplementation on the four major CD4+ populations in human PB; naïve (CCR7+/CD45RA+), effector memory (T_EM_, CCR7-/CD45RA-), central memory (T_CM_, CCR7+/CD45RA-) and terminally differentiated effector memory cells re-expressing CD45RA (TEMRA, CCR7-/CD45RA+).

Baker's yeast beta glucan (BYBG) supplementation did not affect the expected response of naïve or TEMRA CD4+ cells to a bout of strenuous exercise (data not shown), but did change the numbers of effector memory (T_EM_) and central memory (T_CM_) cells in circulation (Figures 5B,C). The number of T_EM_ in circulation after exercise when subjects had been supplemented with BYBG was increased compared to placebo and followed a similar pattern of change over the recovery period as in the placebo condition, but maintained a concentration nearly the same as placebo pre-exercise (Figure 5B). The response of T_CM_ cells after BYBG supplementation was similar to that observed for total CD4+ and CD4+ T_EM_ cells (Figure 5C). The number of circulating T_CM_ cells was elevated before exercise after 10-days of BYBG supplementation and was maintained at a consistent percent increase compared to placebo throughout the recovery period (Figure 5C), similar to the response of the total CD4+ and CD4+ T_EM_ cell populations (Figure 5).

### CD8+ T cells

**Figure 6:**
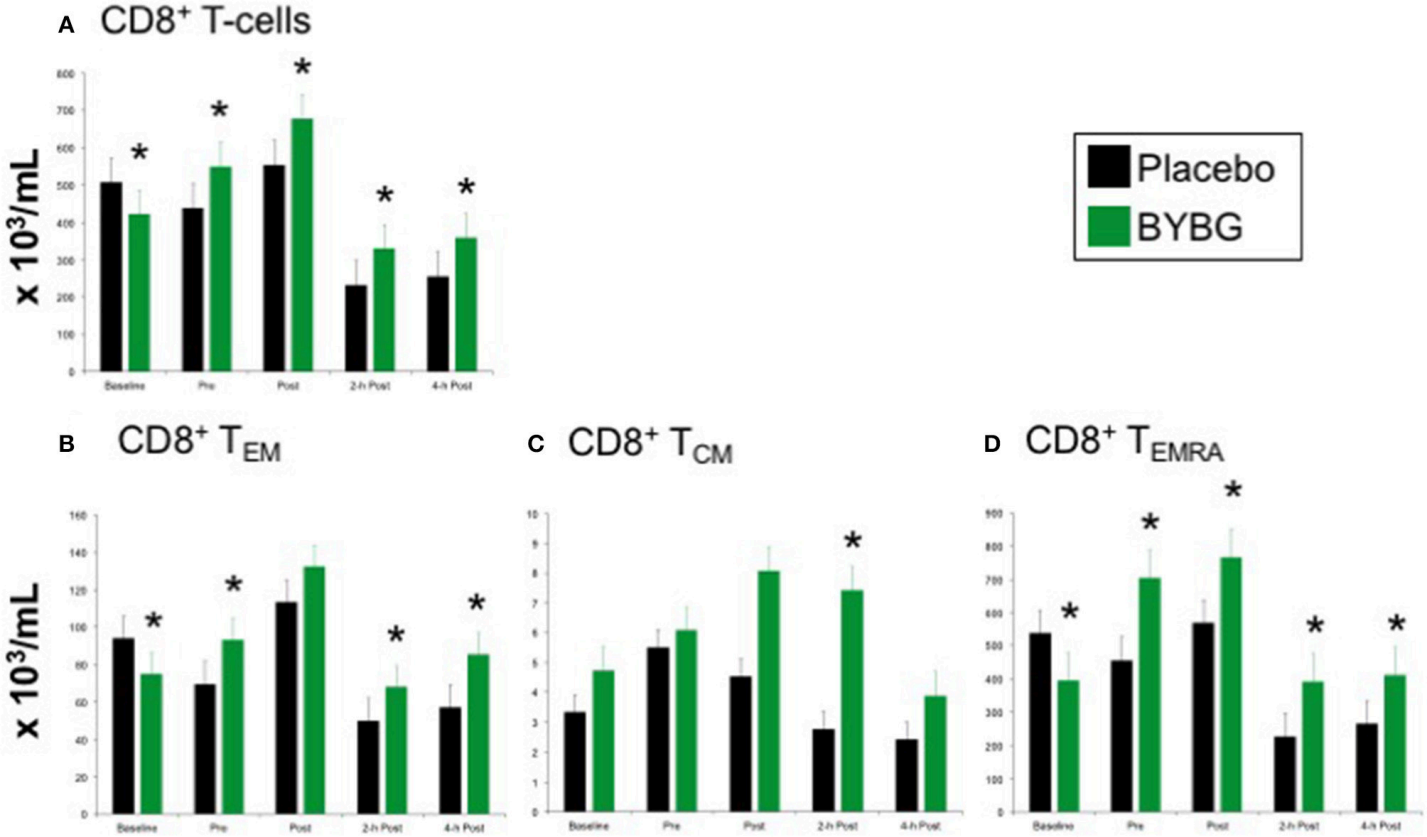
Effect of BYBG on CD8+ T cells. CD8+ T cell concentrations were determined using peripheral blood mononuclear cells (PBMC) isolated from human whole blood samples. PBMCs were analyzed using flow cytometry to determine total (**A**, CD3+/CD8+), T_EM_ (**B**, CCR7-/CD45RA-), and T_CM_ (**C**, CCR7+/CD45RA-), and T_EMRA_ (**D**, CCR7-/CD45RA+) concentrations. Placebo (black bars) and BYBG (green bars) are represented separately and were statistically compared. Values represent the mean ± SEM. ^*^indicates BYBG significantly different than placebo (*p* < 0.05).

The expected response of CD8+ T cells to a bout of strenuous exercise is similar to that described above for CD4+ cells (Nieman et al., 1994; Turner et al., 2010) and was elicited as expected by this exercise protocol (black bars, Figure 6). Supplementation with BYBG increased the number of circulating CD8+ T cells even before exercise stress (Figure 6A). This increased number of total CD8+ T cells compared to placebo was observed immediately after exercise as well as during the first 4 h of recovery (Figure 6A).

Again, PB CD8+ cells were divided into naïve, T_EM_, T_CM_, and TEMRA cells based on CCR7 and CD45RA expression as described above. After BYBG supplementation and before exercise changes in the number of T_EM_ and TEMRA populations were evident (Figures 6B,D). After exercise, T_EM_ cells were increased by consistent percentage compared to placebo at 2- and 4-h post-exercise (Figure 6B). Similarly, CD8+TEMRA cells were increased by a consistent percentage after BYBG supplementation and at all time points before and after exercise (Figure 6D). In contrast to this pattern, CD8+T_CM_ cells were not increased before exercise but did increase during the early period of recovery compared to the placebo condition. Subjects supplemented with BYBG maintained CD8+T_CM_ cells at somewhat increased levels (compared to pre-exercise) through 2-h post-exercise timepoint (Figure 6C).

### Serum cytokines

**Figure 7:**
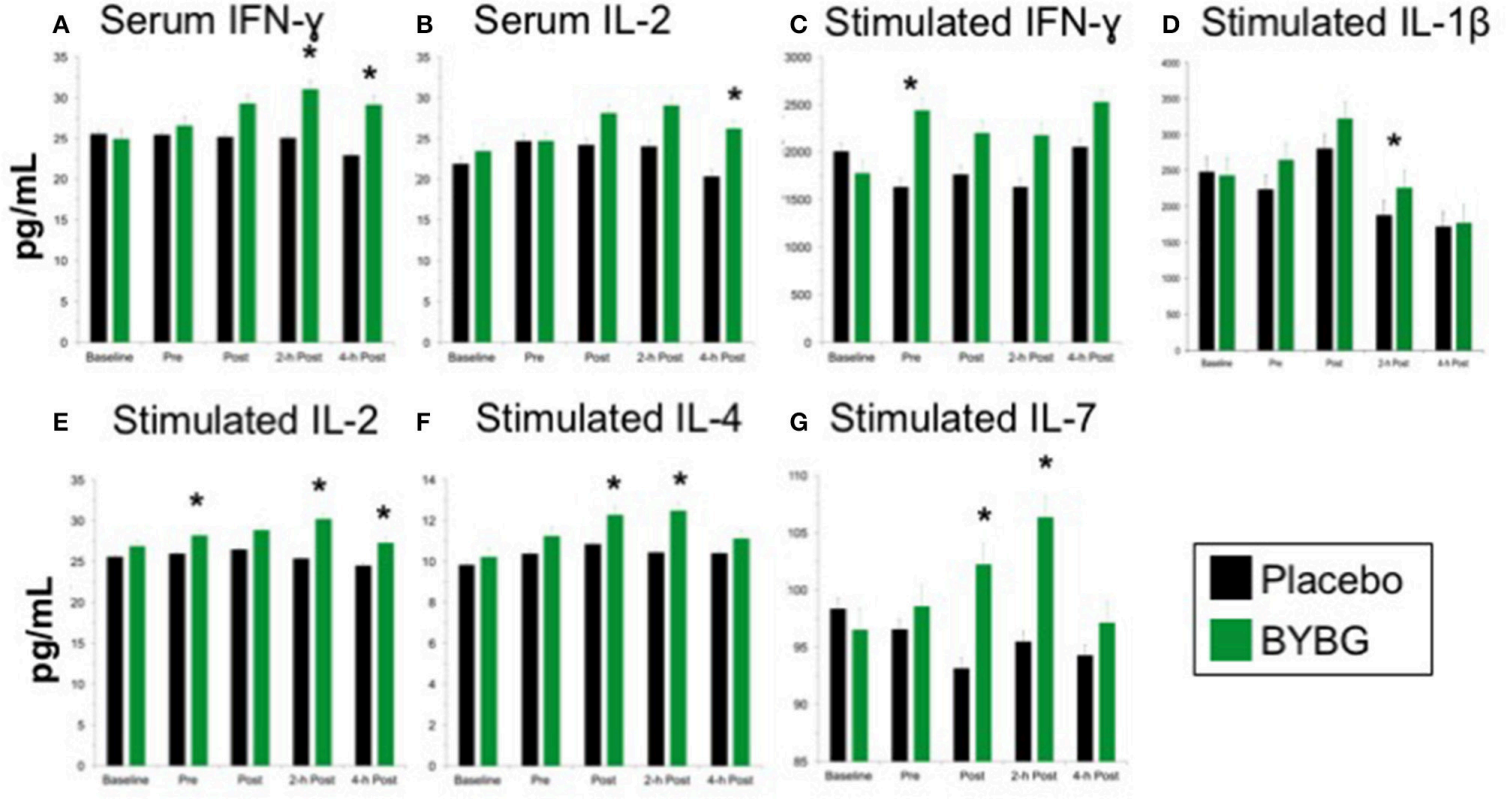
Effect of BYBG on serum and LPS-stimulated cytokines. Cytokine concentrations were determined using a multiplex bead-based assay that was optimized for either low concentration serum (high-sensitivity kit) or tissue culture supernatant (standard sensitivity) samples. All samples were analyzed in duplicate. Of the measured cytokines, significant differences between BYBG and placebo were observed for serum IFN-γ **(A)** and IL-2 **(B)**. Differences were also observed for LPS-stimulated IFN-γ **(C)**, IL-1β **(D)**, IL-2 **(E)**, IL-4 **(F)**, and IL-7 **(G)**. Values represent the mean ± SEM. ^*^indicates BYBG significantly different than placebo (*p* < 0.05).

**Figure 8:**
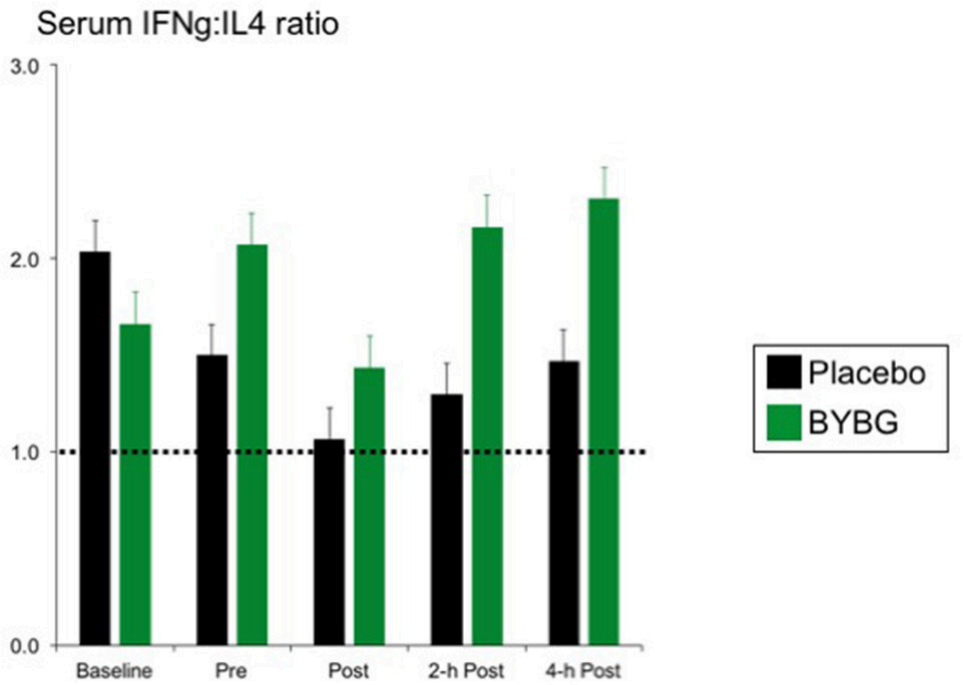
BYBG supplementation shifts the ratio of IFN-γ/IL-4 in serum after exercise toward IFN-γ. The ratio of the serum concentrations of IFN-γ and IL-4 was calculated on an individual donor basis and the average of all donors was computed and is reported as the mean response ± SEM.

Baker's yeast beta glucan (BYBG) supplementation significantly increased serum IFN-γ (*P* = 0.010) and approached significance for IL-2 (*P* = 0.050) (Figures 7A,B). No other serum cytokines (IL-4, IL-5, IL-7) or chemokines (IL-8) measured demonstrated a significant main effect (treatment) or interaction effect. However, the traditional exercise response was observed in a manner consistent with previous findings (data not shown) (Pedersen and Bruunsgaard, 1995; Mitchell et al., 2002; McFarlin and Mitchell, 2003; McFarlin et al., 2004; Simpson et al., 2009; Carpenter et al., 2013). It has been proposed that strenuous exercise increases susceptibility to infection in part due to a disruption of Th1/Th2 T cell homeostasis. Characterization of a T cell as Th1 or Th2 is based on both transcription factors expressed and the cytokine repertoire of the cell. These assays were beyond the scope of the study. However, it was possible to examine the ratio of the Th1-associated cytokine IFN-γ to the Th2-associated cytokine IL-4 in serum. It has been demonstrated in recreational marathon runners that the ratio of IFN-γ:IL-4 mRNA as well as similar changes in other Th1 or Th2-associated genes is skewed after exercise toward a Th2 response (Xiang et al., 2014). In the current study although no changes in the quantity of IL-4 present in serum was observed, serum IFN-γ was increased after BYBG supplementation. This resulted in modulation of the Th1/Th2 cytokine ratio in serum toward a Th1(IFN-γ) cytokine profile (Figure 8). The changes did not reach statistical significance but did show a trend toward significance at 4-h (*p* = 0.092).

### Stimulated cytokine production

Measurement of *in vitro* cytokine responses to LPS-stimulation is a proxy assay for bacterial infection in human systems. We used LPS-stimulation of whole blood to determine whether BYBG supplementation affects the PBMC response to molecular components of a bacterial infection. BYBG supplementation increased secretion of IFN-γ (*P* = 0.038), IL-2 (*P* = 0.001), IL-4 (*P* = 0.0459), and IL-7 (*P* = 0.034) in LPS-stimulated whole blood cultures (Figures 7C–G). Neither BYBG treatment or interaction effects were observed for IL-1β, TNF-α, IL-10, MCP-3, MIP-1α, or MIP-1β; however, the traditional exercise response was observed in a manner consistent with previous findings from our laboratory and others (data not shown).

## Discussion

The present study represents a continuation of research from our laboratory which has sought to identify and characterize naturally occurring substances with potential to prevent post-exercise immunosuppression (Carpenter et al., 2013; McFarlin et al., 2013, 2015, 2016). Our previous research was the first to demonstrate the specific immune system effects by which BYBG prevented post-exercise immunosuppression (Carpenter et al., 2013; McFarlin et al., 2013). Our previous and current findings are consistent with what other laboratories have reported regarding actions of BYBG (Rowe et al., 2009; Talbott and Talbott, 2009). It is important to note that although beta glucan can be derived from a variety of sources (Pharmacopiea, 2011; Walsh et al., 2011), consistent results have only been reported with respect to beta glucan purified from baker's yeast. It is therefore important to be cognizant of the source of the beta glucan when comparing studies. Also, an individual's physical activity status greatly effects how their body responds to exercise stress (Pedersen and Bruunsgaard, 1995; Walsh et al., 2011). Thus, prior to the present study it was not known how accurately results from previous BYBG studies using trained individuals would translate to a general population, who tends to have a lower physical fitness level. We hypothesized that the effect of BYBG supplementation would be similar for individuals of average physical fitness after a bout of strenuous exercise although potentially less pronounced. The key findings of the present study support this hypothesis.

We observed increased concentration of peripheral blood monocytes at 2-h and 4-h after exercise compared to placebo. When the kinetics of the exercise response were examined, subjects on placebo experienced on average a 23 and 29% drop in circulating monocytes at 2- and 4-h post exercise, respectively compared to pre-exercise. In our previous study we noted a 17% decline in total monocyte concentration at 2-h with placebo suggesting that the overall decline in monocyte concentration in the present study was more severe. Interestingly, the BYBG effect in the current study was the same as that observed in our previous study, despite the larger net decline in monocyte concentration post-exercise (Carpenter et al., 2013). All subjects experienced a small magnitude increase in monocyte concentration immediately following exercise (POST), but when the exercise was preceded by BYBG supplementation the result was no net change in circulating monocyte concentration during recovery compared to pre-exercise (2- and 4-h). One factor that may have contributed to the change in monocyte trafficking behavior is the reduction in CD38 expression observed on classic monocytes after BYBG supplementation. CD38 is known to be an important part of the trafficking and adhesion machinery that affects extravasion of leukocytes from the vessels (Dianzani et al., 1994; Musso et al., 2001). The reduction in CD38 expression may be permissive for increased monocyte circulation after exercise which may result in increased immunosurveillance. This interpretation is consistent with other research which has demonstrated that monocyte adhesion molecule expression determines migration capacity into inflamed or infected tissue compartments (Liberek et al., 2004; Iijima et al., 2011).

This study extended our previous observations by exploring whether there were phenotypic changes to either classic or non-classic monocytes associated with the BYBG effect. We observed an increase in CD86 expression on non-classic monocytes at all times after BYBG supplementation which may indicate a more robust capacity of the non-classic monocytes to provide co-stimulation to T-cells in response to an antigenic challenge. Although TLR2 expression was decreased on both classic and non-classic monocytes, an overall increase in TLR4 expression on non-classic monocytes was observed. The change to TLR4 expression levels may be a consequence of increased circulating IFN-γ (Bosisio et al., 2014). Pattern recognition receptors have a key role in the immune system's danger response, which can be activated by either exogenous (i.e., PAMP) or endogenous (i.e., DAMP) signals. TLR4 has been linked to the regulation of post-exercise inflammation following resistance exercise (McFarlin et al., 2004) and thus, the observed response may be indicative increased immune surveillance.

To our knowledge, the present study is the first published report that BYBG supplementation modulates post-exercise T-cell response. T-cell response to a strenuous bout of exercise has been reported to be similar to that of monocytes, namely a transient increase in concentration post-exercise followed by a decrease below pre-exercise during the recovery period (Kruger and Mooren, 2007). We observed a significant increase in the number of total circulating CD4+ and CD8+ cells prior to exercise after 10-days of BYBG supplementation. Of note, the exercise response was preserved in these cell populations in BYBG-supplemented subjects with similar percent decreases from pre-exercise levels as in placebo during the 4-h recovery period. This suggests that the increased number of circulating CD4+, CD4+ T_CM_, and CD8+ T_EM_ and CD8+ T_EMRA_ cells present in BYBG-supplemented subjects before exercise is likely responsible for the increased number of these cells observed circulating post-exercise and during recovery.

In contrast, the effect of BYBG supplementation on CD8+ T_CM_ cells was observed only after exercise and on post-exercise circulation dynamics. This increased presence of CD8+ T_CM_ cells in peripheral blood has been suggested to be an indicator of increased immune surveillance (van Aalderen et al., 2015). The difference observed in the post-exercise response of T_CM_ cells from T_EM_ and TEMRA cells suggests that BYBG supplementation may exert a specific effect on CD8+ T_CM_ cells or the processes that regulate their egress from secondary lymphoid tissues. The present study aimed to characterize the T-cell subset response to strenuous exercise; however, additional research will be required to investigate the mechanisms responsible for these observations.

It has been reported that suppression of monocyte capacity to produce Th1 cytokines can affect the immune response to peripheral tissue infection (Iijima et al., 2011). It is reasonable to speculate that modulation of cytokine production may be related to the efficacy of systemic immune surveillance. Therefore, in addition to investigating the profile of circulating immune cells, we examined the quantity of cytokines present both in blood and secreted by effector cells after LPS-stimulation. The use of the *in vitro* LPS stimulation assay represents a useful proxy measure for the response to a bacterial challenge (McFarlin et al., 2004, 2006, 2007b; Walsh et al., 2011). This assay measures predominantly the response of monocytes (via CD14/TLR4 mechanism) to a bacterial challenge while serum cytokine concentrations reflect the response of a variety of leukocyte types (Walsh et al., 2011; McFarlin and Venable, 2014; McFarlin et al., 2015).

Previously we have reported an increase in serum IFN-γ in highly trained individuals following exercise when supplemented with BYBG (Carpenter et al., 2013). In the present study BYBG supplementation was also associated with an increase serum IFN-γ concentration post-exercise in lower fitness subjects. This consistent effect of BYBG on serum IFN-γ concentration across study populations strengthens confidence in this observation. It suggests that BYBG affects pathways that regulate IFN-γ production in a manner that is not dependent on physical fitness level. Likewise, when the cytokine secretion potential from individuals of average fitness was assessed by LPS-stimulation, we observed an increase in the capacity to secrete IFN-γ in BYBG-supplemented subjects. These observations demonstrate that previously reported responses to BYBG (Carpenter et al., 2013; McFarlin et al., 2013) are not specific to a highly physically trained population and can be generalized to individuals of lower physical fitness. One mechanism of action is BYBG binds to neutrophils and increases their recognition of pathogen associated molecular patterns (PAMP) (Hong et al., 2004; Qi et al., 2011). Based on our current and previous findings, we speculate that BYBG may exert similar “priming” effects on monocytes, such that capacity to produce Th1 cytokines is enhanced. Our observed increase in monocyte TLR4 expression supports the hypothesis that monocytes have been “primed” by BYBG to respond more effectively to bacterial challenge. Enhanced monocyte response capacity may partially explain previously reported improvements in upper respiratory tract infection (URTI) outcomes in marathon runners (Talbott and Talbott, 2009; McFarlin et al., 2013) and wilderness firefighters (Harger-Domitrovich et al., 2008).

In summary, the present study demonstrated that oral BYBG supplementation was associated with increased circulating monocyte concentration, altered monocyte cell-surface receptors, increased T-cell concentration, increased serum IFN-γ, and an increase in LPS-stimulated production of IFN-γ and IL-2. Based on the current literature the observed immune responses would be consistent with an increased ability to respond to antigenic challenge. When combined with our previous studies (Carpenter et al., 2013; McFarlin et al., 2013), it appears that the systemic effect of oral BYBG supplementation was independent of physical fitness level. This supports the hypothesis that BYBG supplementation supports the post-exercise immune system in individuals with a wide range of physical fitness levels. There are many kinds of physical stress beyond structured exercise, so future research may seek to apply this supplementation paradigm to individuals who work in stressful, physically demanding jobs (i.e., law enforcement officers, public safety officers, and soldiers).

## Author contributions

BM, AV, KC, AH, and SO contributed to the design, collection, analysis, and interpretation of the data represented in this manuscript. They all reviewed and assisted in editing the final manuscript. SO specifically contributed to the statistical analysis since he is a trained biostatistician.

### Conflict of interest statement

The authors declare that the research was conducted in the absence of any commercial or financial relationships that could be construed as a potential conflict of interest.
